# 1258. Consulting Colleagues: Increasing Role of Advanced Practice Providers in Inpatient ID Consultation

**DOI:** 10.1093/ofid/ofad500.1098

**Published:** 2023-11-27

**Authors:** Reinaldo Perez, Michael E Yarrington, Connor R Deri, Michael J Smith, Jillian E Hayes, Rebekah Wrenn, Rebekah W Moehring

**Affiliations:** Duke University, Durham, North Carolina; Duke University Health System, Durham, North Carolina; Duke University, Durham, North Carolina; Duke University, Durham, North Carolina; Duke University Hospital, Durham, North Carolina; Duke University, Durham, North Carolina; Duke University, Durham, North Carolina

## Abstract

**Background:**

Advanced practice providers (APPs) have taken on increasing roles as primary team members in acute care hospitals. Little is known about variable consulting practices across provider types. Here we describe longitudinal trends in infectious diseases (ID) consultation by attributed provider type in 3 hospitals.

**Methods:**

We performed a retrospective time series analysis of ID consultation from July 2015 to June 2022 to investigate the changes in requesting provider type at 3 hospitals: a major university hospital and 2 community hospitals. ID consultation rates were based upon new consult orders placed into the electronic health record (EHR). Provider type was categorized by type of ordering clinician: physicians, trainees (residents, fellows and medical students), and APPs (nurse practitioners, physician assistants, and nurse anesthetists). We evaluated the number of ID consult orders over time to assess quarterly rate trends. Then, we calculated the percent of ID consults attributed to each provider group. We used multinomial logistic regression to measure changes in ID consults across the clinician groups over time using physicians as the referent.

**Results:**

We observed an overall increase in rate of ID consultation per 1000 days present by 35% (Table 1, Figure 1). Each hospital had a distinct baseline care model, though all 3 showed increased consultation by APPs (Figure 2, Table 2). This shift was largest at the university hospital, and was a statistically significant increase relative to attending physicians. Trainee proportion of consultation was stable at community hospitals and decreased at the university hospital.

**Figure:**
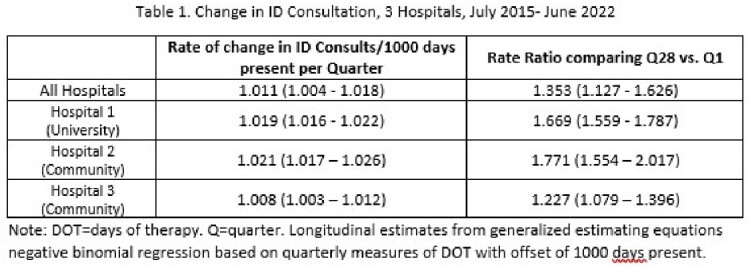

**Figure:**
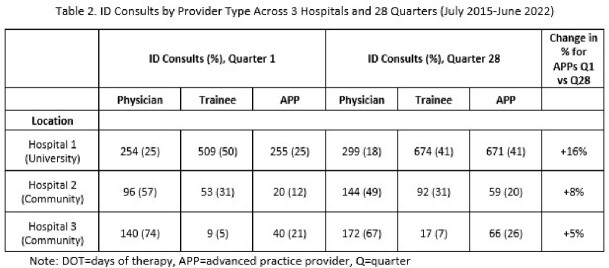

**Figure:**
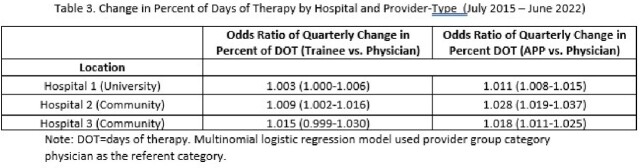

**Figure:**
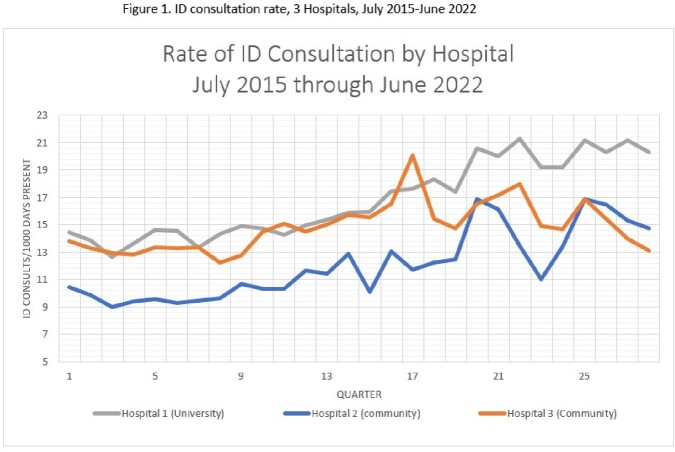

**Figure 2.**
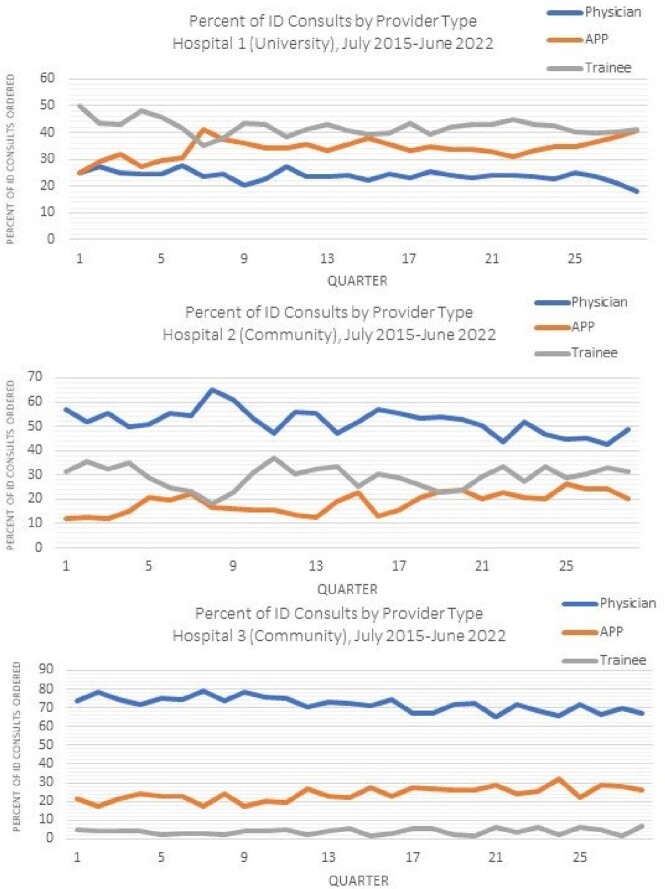
Percent of ID Consults Ordered by Provider Type and Hospital, July 2015 – June 2022

**Conclusion:**

Hospitals had differing baseline patterns of ID consultation attributed to provider groups, but all experienced increases in consults attributed to APPs and increased ID consultation rates over time. Hospital staffing models aiming to increase use of APPs must consider consultation rates as a potential effect and support infectious diseases services accordingly. As research showing the benefits of ID consultations expands and consultation increases, ID providers will need to continue to optimize their interprofessional collaboration.

**Disclosures:**

**Michael J. Smith, M.D., M.S.C.E**, Merck: Grant/Research Support|Pfizer: Grant/Research Support **Rebekah W. Moehring, MD, MPH, FIDSA, FSHEA**, UpToDate, Inc.: Author Royalties

